# A Type III Monteggia Injury with Ipsilateral Fracture of the Distal Radius and Ulna in a Child: Case Report Followed for 21 Years

**DOI:** 10.1155/2018/1876075

**Published:** 2018-06-21

**Authors:** Takeshi Inoue, Makoto Kubota, Keishi Marumo

**Affiliations:** Department of Orthopaedic Surgery, Jikei University School of Medicine, 3-25-8 Nishishinnbashi, Minato-ku, Tokyo 105-8461, Japan

## Abstract

Bado type III Monteggia injuries complicated by ipsilateral forearm fractures are extremely rare. We report a case of a 6-year-old boy who sustained such an injury after falling from the top of a 3 m climbing pole. He was diagnosed with a Bado type III Monteggia fracture and forearm fractures. Manual reduction was attempted on the day of injury. However, because it was difficult to maintain the reduction of the radial head, open and percutaneous procedures were performed to reduce and fixate the fractures with Kirschner wires. The postoperative course was favorable. Twenty-one years later, the patient, now 27 years old, had no decreased range of joint motion or problems with activities of daily living. The fracture morphology observed in this case is rare, and this is the only case for which long-term follow-up has been carried out to adulthood.

## 1. Introduction

In pediatric patients, fractures around the elbow and wrist joints are often encountered. However, patients with concomitant fractures of the ipsilateral elbow and wrist joints are rarely seen [1]. Monteggia fracture is a rare fracture that is observed in only 0.4% of all forearm fractures [2]. The condition is named after Giovanni Battista Monteggia, who reported 2 patients with fractures of the proximal third of the ulna with anterior dislocation of the radial head in 1814 [3]. These lesions have most commonly been further classified in accordance with the Bado classification system [4].

Here, we report an extremely rare case of type III Monteggia injury with ipsilateral fracture of the distal radius and ulna in which the patient was followed up for 21 years.

## 2. Case Presentation

**Figure 1:**
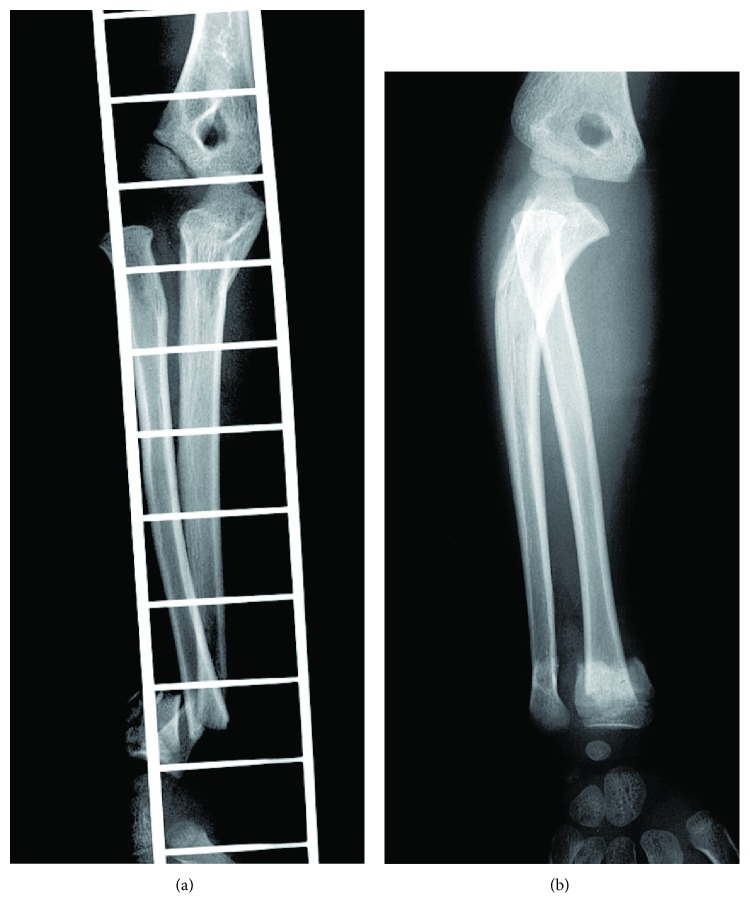
X-rays at first visit. A Bado type III Monteggia fracture and fractures of the distal radius and ulna were observed on the same side. (a) AP view of the elbow joint. (b) Oblique view of the elbow joint.

A 6-year-old boy with no pathological history accidentally fell from the top of an approximately 3 m climbing pole and injured his right extended elbow and wrist joint. Due to pain and deformity in the right elbow and wrist joints, he visited our hospital. Swelling and a dinner fork deformity of the right wrist joint and pronounced swelling of the right elbow joint were observed. No skin damage was observed. No findings of nerve injury or arterial injury were obtained in the right upper limb. Radiography revealed lateral dislocation of the radial head, a fracture of the proximal ulnar metaphysis, and mild bending deformation at the fracture site. In addition, fractures of the distal radius and ulna, as well as dorsal displacement of the distal fragment, were seen (Figure 1). Thus, the patient was diagnosed with Bado type III Monteggia injury with ipsilateral fracture of the distal radius and ulna.

**Figure 2:**
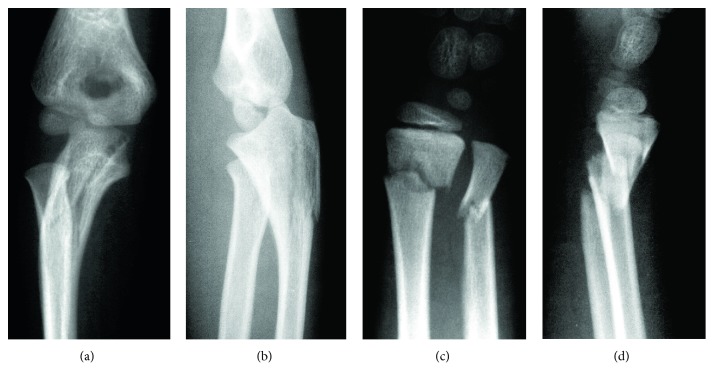
X-rays images after manual reduction. (a) AP view of the elbow joint showing subluxation of the radial head. (b) Lateral view of the elbow joint. (c) Front view of the wrist joint showing dislocation of the distal radius. (d) Lateral view of the wrist joint showing dislocation of the distal radius.

**Figure 3:**
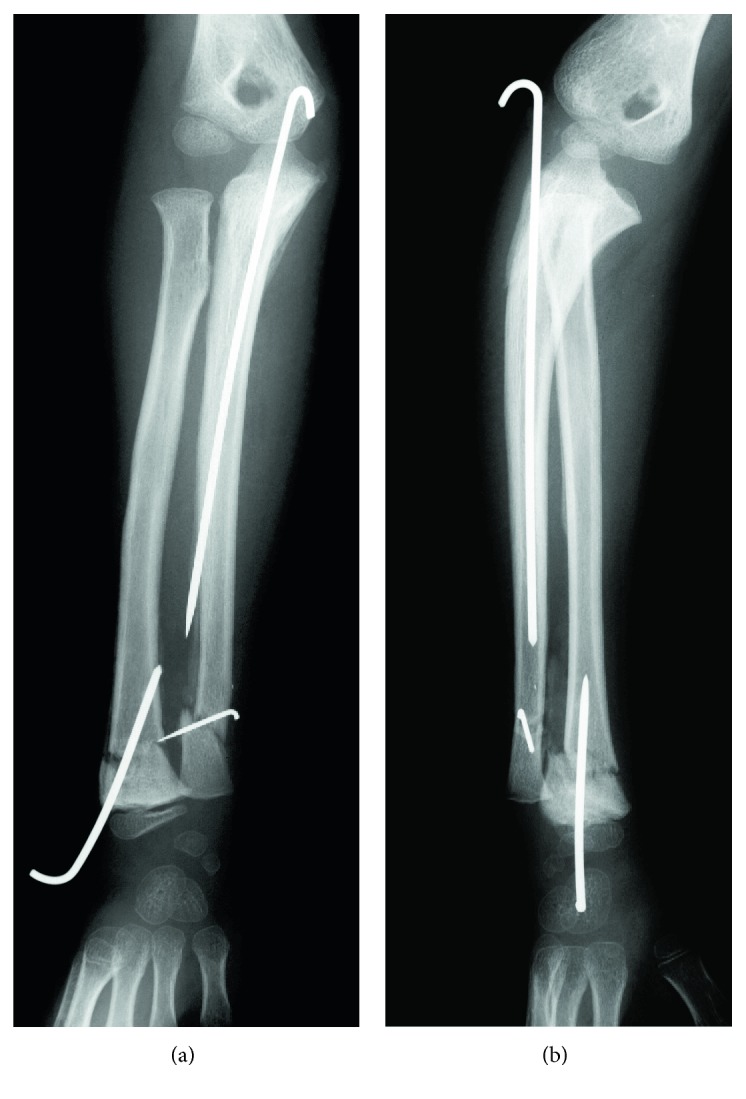
X-rays postoperation. (a) AP view. (b) Lateral view.

Manual reduction under nerve block was attempted on the day of injury. However, because it was difficult to maintain the reduction of the radial head, as shown in Figure 2, open reduction and percutaneous procedures were performed under general anesthesia. A Kirschner wire was inserted, percutaneously, from the olecranon into the ulnar diaphysis. When the Kirschner wire was in place, the dislocation of the radial head immediately showed good reduction. Further, open reduction and fixation of the fractured distal radius and ulna were performed with Kirschner wires (Figure 3). A long-arm cast was used for external fixation with the elbow in 90° flexion and the forearm in an intermediate position.

**Figure 4:**
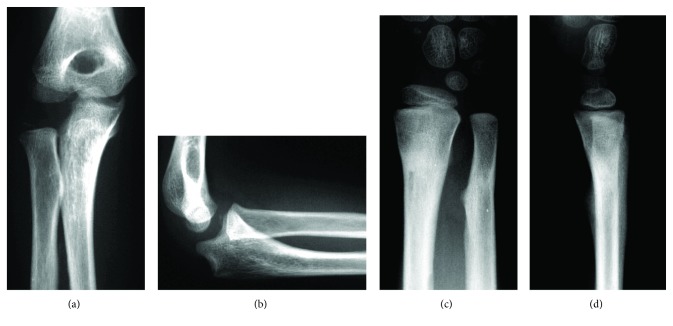
X-rays 3 months after surgery. (a) Front view of the elbow joint. (b) Lateral view of the elbow joint. (c) Front view of the wrist joint. (d) Lateral view of the wrist joint.

Two weeks after surgery, callus formation at the fractured bone was observed. Therefore, the cast was removed, and range of motion (ROM) exercises of the elbow and wrist joints were initiated. Since bone union was achieved at 6 weeks postsurgery, the Kirschner wires were removed. Pain, ROM limitation, and lateral instability were not observed in the elbow or wrist joints at 3 months after surgery. Additionally, plain radiographs taken at the same time showed a radially convex curvature at the proximal portion of the ulna and lateral subluxation of the radial head (Figure 4). However, a gradual correction in the outward displacement of the radial head was observed during the 3-year follow-up.

**Figure 5:**
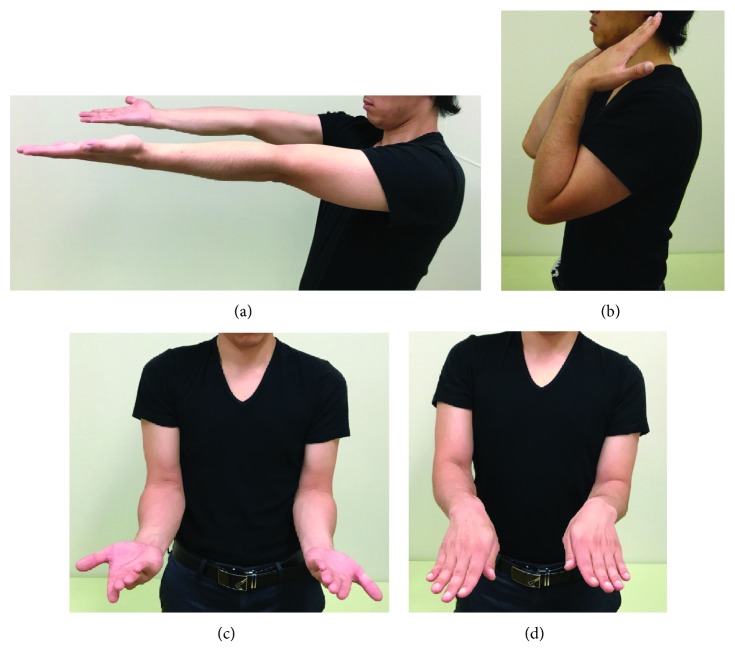
Twenty-one years after surgery. (a) Extended elbow. (b) Flexed elbow. (c) Forearm supination. (d) Forearm pronation.

**Figure 6:**
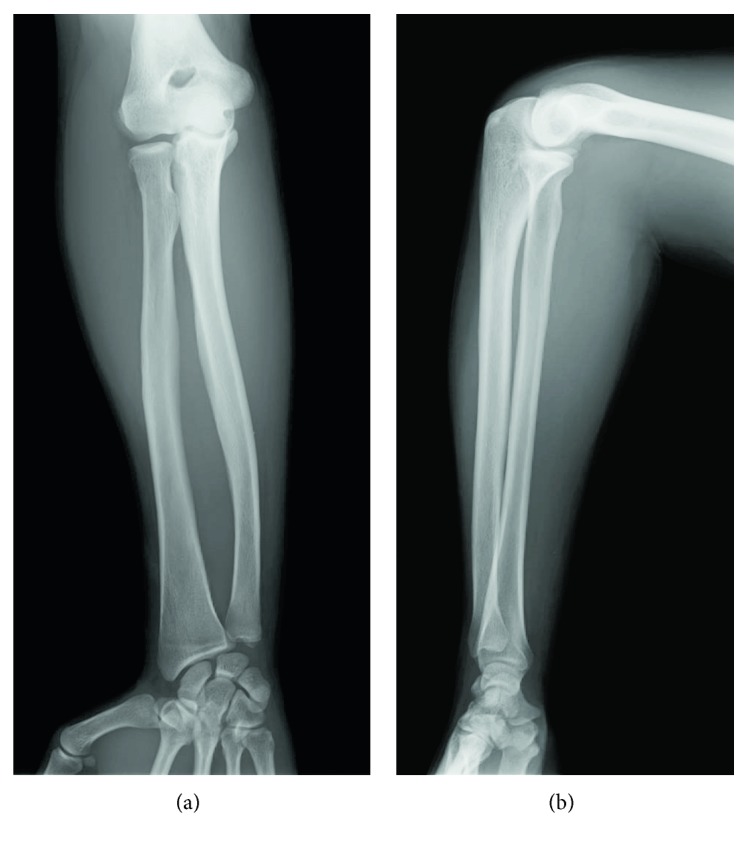
X-rays 21 years after surgery. (a) Front view. (b) Lateral view. The compatibility of the radiocapitellar joint was good, and no malunion was observed.

Twenty-one years after surgery, the patient returned to our hospital for another disorder. At that time, we obtained informed consent to perform an examination and take radiographs of the previous Monteggia injury. Neither spontaneous pain, pain during exercise, tenderness, nor ROM asymmetry were observed (Figure 5). The biocompatibility of the radiocapitellar joint was good, and no malunion was found in the distal radius and ulna (Figure 6). The patient reports that he has been working as a computer programmer and performs weight training as a hobby without limitations.

## 3. Discussion

Nerve injuries, vascular injuries, compartment syndrome, and ipsilateral fractures of the forearm, in the early stages of the injury, as well as redislocation and malunion of the ulna fracture site, in the late stages, have been reported as complications of Monteggia fractures [5]. Fractures of the forearm, including distal radius fractures [6–8], have rarely been reported in association with Monteggia fractures, as documented by Letts et al. [9] and Olney and Menelaus [5]. They found that only one out of 33 or 2 out of 102 patients with Monteggia fractures exhibit associated fractures [5, 9]. To our knowledge, beyond these cases, only 3 cases of Monteggia fractures with ipsilateral fracture of the distal radius and ulna have been reported, as in the present case [10–12].

The observation period in all previous reports of Monteggia injury with ipsilateral forearm fractures has been 2 years or less [6–8, 10–14], except for a 6-year follow-up reported by Biyani [1]. This is the only case wherein long-term postoperative follow-up evaluation was feasible until adulthood.

There is no established theory of the pathogenesis of Monteggia fracture. In addition, it is difficult to infer the mechanisms of double fractures, as in this case. However, Sinha et al. [12] reported a similar case of an affected child who fell with their forearm pronated and the wrist dorsiflexed, which resulted in distal radius and ulna fractures. The impact from the fall was subsequently transmitted to the elbow, which was in valgus extension, and resulted in dislocation of the radial head and fractures in the proximal end of the ulna [12].

While treating a Monteggia fracture, examination of both the cubital joint and the wrist joint is important because a fracture of the distal forearm may also occur, as in this case [10]. Since a child who has sustained such an injury may be experiencing pain and/or anxiety and therefore unable to sufficiently express himself, the presence or absence of any swelling, deformities, abrasions possibly due to direct force, and ROM limitation should be carefully examined. It is important to perform accurate radiography in 2 planes (i.e., frontal and lateral views) [6, 10]. Since Monteggia fractures in children are mostly incomplete greenstick fractures, reduction and maintenance are easy to perform. Therefore, conservative treatment can often be employed in cases with timely diagnosis [15, 16]. However, since it was difficult to perform manual reduction owing to a double fracture of the ulna, surgery was the only treatment option available in the presented patient. Nevertheless, to obtain good treatment results, it is important to accurately diagnose and promptly treat the condition [15, 16].
